# Synthesis of ZnO Hierarchical Structures and Their Gas Sensing Properties

**DOI:** 10.3390/nano9091277

**Published:** 2019-09-07

**Authors:** Chao Fan, Fazhe Sun, Xiaomei Wang, Zuzhen Huang, Mina Keshvardoostchokami, Parveen Kumar, Bo Liu

**Affiliations:** 1 Laboratory of Functional Molecular and Materials, School of Physics and Optoelectronic Engineering, Shandong University of Technology, Zibo 255000, China; fc17509190@outlook.com (C.F.); zuzhen_huang@163.com (Z.H.); minak@sdut.edu.cn (M.K.); kumar@sdut.edu.cn (P.K.); 2 School of Material Science and Engineering, Shandong University of Technology, Zibo 255000, China; 3 Analysis and Testing Center, Shandong University of Technology, Zibo 255000, China; fazhesun@163.com

**Keywords:** hierarchical structures, electrospinning, H_2_S, hydrothermal, gas sensing

## Abstract

Firecracker-like ZnO hierarchical structures (ZnO HS1) were synthesized by combining electrospinning with hydrothermal methods. Flower-like ZnO hierarchical structures (ZnO HS2) were prepared by a hydrothermal method using ultrasound-treated ZnO nanofibers (ZnO NFs) as raw material which has rarely been reported in previous papers. Scanning electron microscope (SEM) and transmission electron microscope’s (TEM) images clearly indicated the existence of nanoparticles on the ZnO HS2 material. Both gas sensors exhibited high selectivity toward H_2_S gas over various other gases at 180 °C. The ZnO HS2 gas sensor exhibited higher H_2_S sensitivity response (50 ppm H_2_S, 42.298) at 180 °C than ZnO NFs (50 ppm H_2_S, 9.223) and ZnO HS1 (50 ppm H_2_S, 17.506) gas sensors. Besides, the ZnO HS2 sensor showed a shorter response time (14 s) compared with the ZnO NFs (25 s) and ZnO HS1 (19 s) gas sensors. The formation diagram of ZnO hierarchical structures and the gas sensing mechanism were evaluated. Apart from the synergistic effect of nanoparticles and nanoflowers, more point–point contacts between flower-like ZnO nanorods were advantageous for the excellent H_2_S sensing properties of ZnO HS2 material.

## 1. Introduction

Hydrogen sulfide (H_2_S) is a flammable, explosive and harmful gas [1]. Long-time exposure to low concentrations of H_2_S atmosphere could cause human irritation in the nasal cavity and eyes, and in high concentrations, an H_2_S atmosphere, even for short-time could cause humans to die [2,3]. Therefore, exploring gas sensors for precise detection of H_2_S gas is becoming an urgent research project.

Recently, 3D hierarchical structures have been extremely appealing due to their unique structures and better gas sensing performance than 1D and 2D materials [4,5,6]. Meanwhile, metal oxide (MO) materials gas sensors, such as ZnO [7,8,9], CuO [10,11], In_2_O_3_ [12,13], WO_3_ [14,15], CeO_2_ [16], SnO_2_ [17,18], etc., have attracted extensive attention. Besides, MO materials exhibit outstanding sensing properties in the detection of H_2_S gas [19,20,21,22]. Kaur et al. [23] prepared In_2_O_3_ whiskers by the carbothermal method and the In_2_O_3_ whiskers exhibited excellent sensing properties toward H_2_S gas. Li et al. [24] reported hierarchical flower-like CuO synthesized by a hydrothermal method, which exhibited high sensitivity and selectivity toward H_2_S. Nakla et al. [25] synthesized flower-like SnO_2_ nanowires through a high current heating method and the SnO_2_ nanowire sensor showed good H_2_S sensing properties. Among these MO materials, zinc oxide (ZnO) has been developed for the detection of toxic gases, owing to its wind band gap (3.37 eV) [26], big exciton binding energy (60 meV) [27] and excellent chemical stability [28]. Various methods have been developed and applied to prepare ZnO nanomaterials, including thermal evaporation electrodeposition [29], physical vapor deposition [30], the hydrothermal method [31] and the solvothermal method [32]. Among these methods, the hydrothermal method has been widely used in the preparation of nanostructures, owing to its advantages of low costs, a low reaction temperature and easy process control. At the current stage, ZnO hierarchical structures have been created and applied for the detection of the harmful gases. Zhu et al. discovered that ZnO hierarchical nanoflower assembly by nanosheets exhibited superior ethanol gas-sensing performances than those of nanoparticles and nanoplates [33]. ZnO hierarchical structure was also considered to be a potential material for detecting low-concentrations of harmful gases [34,35]. ZnO hierarchically structured materials are expected to have excellent H_2_S gas sensing properties. Liu et al. reported the ZnO hierarchical porous structure exhibited excellent sensing performance toward H_2_S, owing to its higher porosity and surface area [36]. However, flower-like ZnO was generally fabricated by the assistance of surfactant [37,38], it is still a challenge to synthesize hierarchical ZnO structures in low-temperatures without using surfactants and other additives.

As far as we know, the preparation of flower-like ZnO hierarchical structures by the hydrothermal method using ultrasound-treated ZnO nanofibers (NFs) as raw material has been rarely reported. Although serval researchers have synthesized ZnO materials with similar morphologies [39,40,41], the gas sensing performances of firecracker-like ZnO hierarchical structures have also been rarely studied. In this work, ZnO nanofibers and ZnO hierarchical structures with firecracker and flower morphologies were prepared and their gas sensing performances were examined in detail. The gas sensing tests showed that all fabricated ZnO materials have a high selectivity toward H_2_S gas while flower-like ZnO hierarchically structured material achieved the best gas sensing performance toward H_2_S at a 180 °C working temperature. Herein, the growth process and gas sensing mechanism of flower-like and firecracker-like ZnO hierarchical structures were also investigated and proposed.

## 2. Materials and Methods

### 2.1. Materials

Absolute ethanol (C_2_H_5_OH, 99.7%), dimethylformamide (DMF, C_3_H_7_NO, 99.5%), zinc acetate dihydrate (Zn(CH_3_COOH)_2_·2H_2_O, 99%), hexamethylenetetramine (HMTA, 99%), zinc nitrate hexahydrate (Zn(NO_3_)_2_·6H_2_O, 99%) and polyvinylpyrrolidone (PVP, 99.5%, M_W_ = 1,300,000) were purchased from Sinopharm Chemical Reagent Co., Ltd. (Shanghai, China). These chemicals were of analytical grades and used without further purification. The electrospinning needle with a 0.33 mm inner diameter was purchased from Suzhou Lanbo Dispensing Needle Co., Ltd. (Suzhou, China).

### 2.2. Synthesis of ZnO NFs

In the typical preparation, 0.2 M Zn (CH_3_COO)_2_·2H_2_O was dissolved in a solution with an equal volume (10 mL each) of N, N-Dimethylformamide (DMF) and absolute ethanol (C_2_H_5_OH). Next, 2.4 g of polyvinylpyrrolidone (PVP) was added to the solution and vigorously stirred for 10 h. In the electrospinning experiment, the applied electrical voltage was set at 12 kV and the distance between the cylinder collector and the syringe needle loaded with precursor solution was maintained at 15 cm. The dosage rate of solution was kept at 0.56 mL/h. After that, the obtained NFs were calcined at 500 °C for 4 h in a furnace with a 1 °C/min heating rate.

### 2.3. Synthesis of Firecracker and Flower Morphological ZnO Hierarchical Structures

Firecracker and flower morphological ZnO hierarchical structures were prepared via electrospinning and hydrothermal methods. In the experiment, 0.280 g HMTA and 0.595 g Zn (NO_3_)_2_·6H_2_O were added to two beakers with 40 mL deionized water (DI water), respectively. Afterward, the aqueous solutions were mixed together and stirred for 5 min. Finally, 0.03 g of the prepared ZnO NFs were put into the solution and agitated for 10 min. The reaction solution was transferred to a 100 mL autoclave and heated at 80 °C for 12 h in the oven. After the temperature of the oven cooled to room temperature, the autoclave was removed. The acquired precipitates were centrifuged and rinsed in the absolute ethanol and DI water three times, respectively. Then the precipitates were dried at 80 °C for 12 h in the oven. At last, ZnO hierarchical structures with firecracker morphology were collected. The difference when preparing flower morphological ZnO hierarchical structures was that the reaction solution with ZnO NFs was sonicated under 100 W power for 2 min before the hydrothermal experiment. The obtained firecracker-like ZnO and flower-like ZnO hierarchical structures are denoted ZnO HS1 and ZnO HS2, respectively.

### 2.4. Characterizations

The phases and compositions of materials were recorded on X-ray diffraction (XRD, Bruker D8 ADVANCE, Karlsruhe, Germany) with Cu Kα radiation (λ = 1.5418Å). The morphology and microstructure of the materials were determined by a scanning electron microscope (SEM, FEI Sirion 200F, USA). High-resolution transmission electron microscopy (HRTEM), transmission electron microscopy (TEM), scanning transmission electron microscopy (STEM) mapping and energy dispersive X-ray (EDX) analysis patterns were obtained by JEOL JEM-2200FS (Tokyo, Japan).

### 2.5. Gas sensing Measurements and Fabrication of Gas Sensors

**Figure 1:**
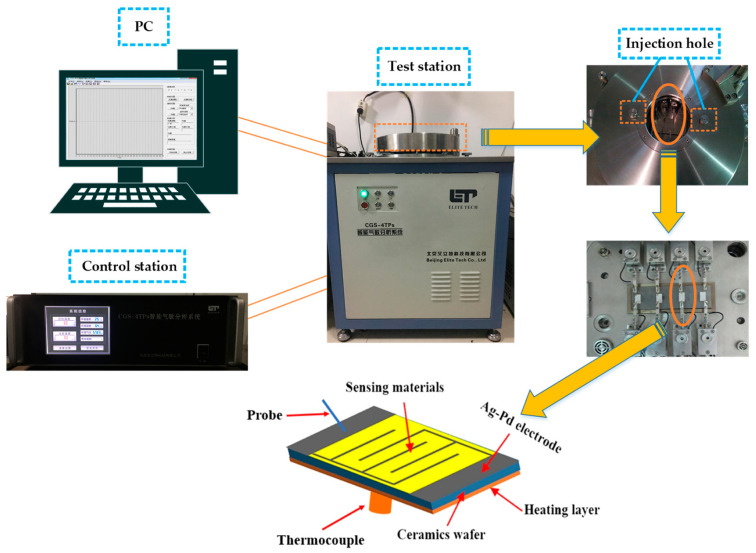
The schematic diagram of the gas sensing analysis system and the composition of a gas sensor.

Figure 1 displayed the gas sensing system used in the gas sensing tests. It can be seen that the gas sensing system is composed of a PC (personal computer), control station and test station. The working temperature of gas sensors can be adjusted from 20 °C to 500 °C by the control station. In this study, the target gases were injected to the inner chamber through injection holes on the test station. Gas sensors were tested on the test station using two silver probes to form a current loop. The data of gas sensing tests were collected on the PC. Additionally, the gas sensor consisted of Ag-Pd electrodes, a heating layer, a ceramics wafer, sensing materials and a thermocouple. In the gas sensors’ manufacturing processes, the prepared materials were mixed together with absolute ethanol and ground by a gridding rod to form a uniform paste. Then the ceramics substrate with electrodes was covered with the paste using a brush. The sensors were annealed at 500 °C for 2 days to improve the stability of sensors. The volume of target gas was calculated and a certain amount of target gas was injected into the test chamber (18 L in volume) using a microsyringe to configure a certain concentration of target gas. The concentration of target gas was controlled by injecting various volumes of gas into the chamber. The gas sensors were examined by a CGS-4TPS intelligent gas sensing analysis system (Beijing Elite Tech Co. Ltd., Beijing, China). The response signal of the gas sensor was defined as Rs = Ra/Rg, where the Ra refers to the resistance of the sensors after it is stabilized in the air and Rg is the resistance of the sensors upon exposure of the target gases. Furthermore, the response time and recovery time were determined as the time to acquire the 90% total resistance change after the target gases were injected, and the time required to reach the 10% total resistance change after the target gases were removed, respectively.

## 3. Results and Discussion

### 3.1. Materials Characterizations

**Figure 2:**
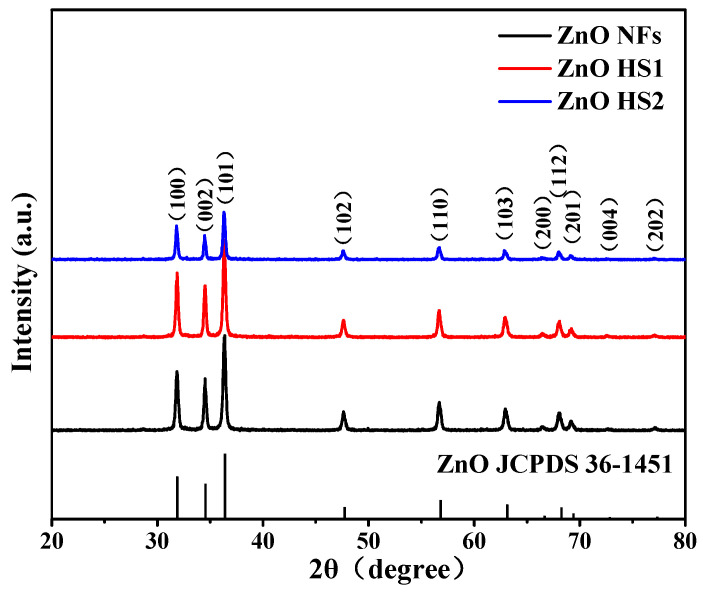
XRD patterns of as-prepared ZnO nanofiber (NF), firecracker-like ZnO (ZnO HS1) and flower-like ZnO (ZnO HS2) materials.

The phase composition of acquired materials was analyzed by XRD. The XRD patterns of ZnO NFs, ZnO HS1 and ZnO HS2 samples were illustrated in Figure 2. All of the diffraction peaks correspond to the hexagonal wurtzite structure ZnO (JCPDS number 36-1451). Moreover, it is worth noting that no impurity peaks are detected and no peaks shift, indicating that the samples were highly pure. The main diffraction peaks at 2θ = 31.9°, 34.5° and 36.3° were indexed to the 100, 002 and 101 planes of hexagonal ZnO, respectively. The shrill peaks revealed the high crystallinity of all prepared samples.

**Figure 3:**
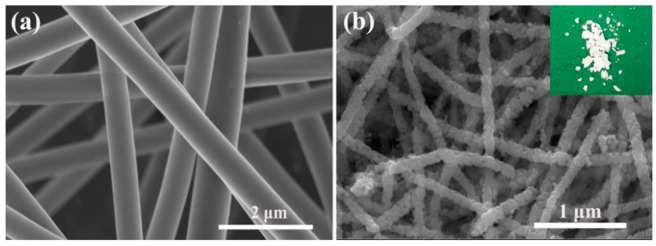
SEM images of as-prepared samples: (**a**) precursor ZnO NFs and (**b**) ZnO NFs. The inset is the digital image of synthesized ZnO NFs material.

The SEM micrographs of precursor and calcined ZnO NFs are displayed in Figure 3, respectively. The synthesized ZnO NFs display white powder morphology, as shown in the inset of Figure 3b. The as-prepared precursor ZnO NFs are smooth and continuous. The ZnO NFs were prepared by annealing the precursor ZnO NFs. The diameter of the precursor ZnO NFs and ZnO NFs were assessed at 430 and 210 nm, respectively. The decrease in diameter is owed to the removal of PVP in precursor ZnO NFs. It can be clearly seen that the ZnO NFs were composed of ZnO nanoparticles, which were tightly bonded.

**Figure 4:**
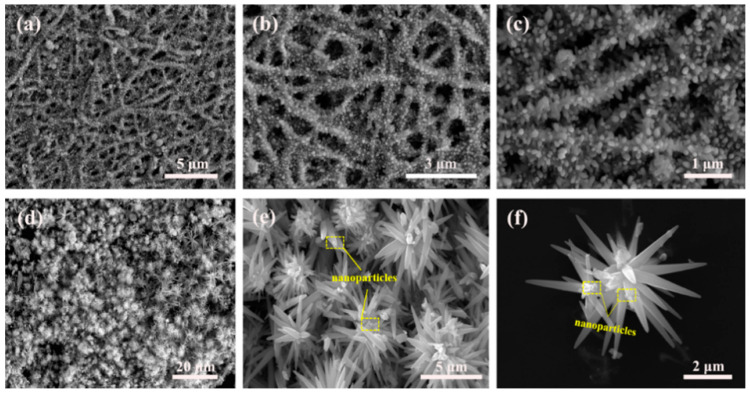
SEM images of as-prepared samples: (**a**) ZnO HS1 in low magnification, (**b**,**c**) ZnO HS1 with high magnification, (**d**) ZnO HS2 in low magnification and (**e**,**f**) ZnO HS2 with high magnification.

Figure 4 shows the SEM pictures of ZnO HS1 and ZnO HS2. Uniform and firecracker-like ZnO hierarchical structures were observed in Figure 4a. Figure 4b,c shows the high magnification of firecracker-like ZnO consisted of ZnO nanofibers and ZnO nanorods. The NFs were in the center and nanorods were uniformly grown on the NFs. The nanorods grown on the NFs were less than 1 μm in length. A number of ZnO HS2 were clearly observed in Figure 4d. ZnO HS2 are made up of needle-shaped nanorods. The high magnification images displayed in Figure 4e,f show that many ZnO nanoparticles were distributed on the flower-like ZnO. These nanoparticles were not the nuclei involved in the formation of flower-like ZnO hierarchical structures.

**Figure 5:**
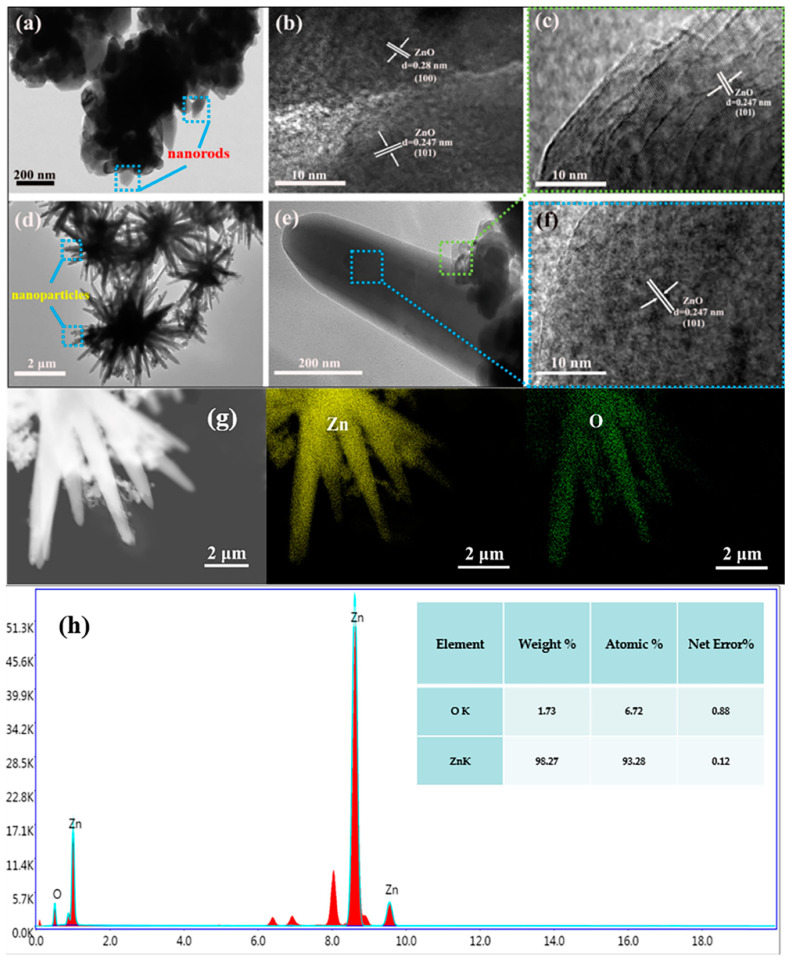
TEM images of as-prepared samples: (**a**) ZnO HS1 with low magnification, (**b**) ZnO HS1 with high magnification, (**d**) ZnO HS2 with low magnification, (**e**) ZnO HS2 with high magnification and (**c**,**f**) HRTEM images of the nanoparticle and nanorod on the ZnO HS2, (**g**) STEM mapping image of ZnO HS2 material and (**h**) EDX analysis of the ZnO HS2 material.

Figure 5 represents the TEM, HRTEM, scanning transmission electron microscopy (STEM) mapping and EDX analysis data of the as-prepared ZnO HS1 and ZnO HS2. As shown in Figure 5a, the firecracker-like ZnO structure material was revealed by the low-magnification TEM images. ZnO nanorods were clearly observed from the image. Figure 5b displays a high magnification TEM image of the firecracker-like ZnO. By measurement, the lattice spacings were determined to be 0.28 nm and 0.247 nm, which were indexed to 100 and 101 planes of hexagonal wurtzite structure of ZnO, respectively. Figure 5d displays the low magnification TEM image of ZnO HS2. Many ZnO nanoparticles were distributed on the ZnO HS2. The lattice spacing of nanoparticles and nanorods were measured to be 0.247 nm in both the cases, as shown in Figure 5c,f. Figure 5g shows the STEM mapping image of ZnO HS2 material. The image indicates that the existence of Zn and O elements of ZnO HS2 material. EDX analysis results of ZnO HS2 were shown in Figure 5h. The results reveal that the ZnO HS2 material had high purity and had no impurity phases. In addition, these results were consistent with the findings of XRD.

### 3.2. Growth Mechanism

**Figure 6:**
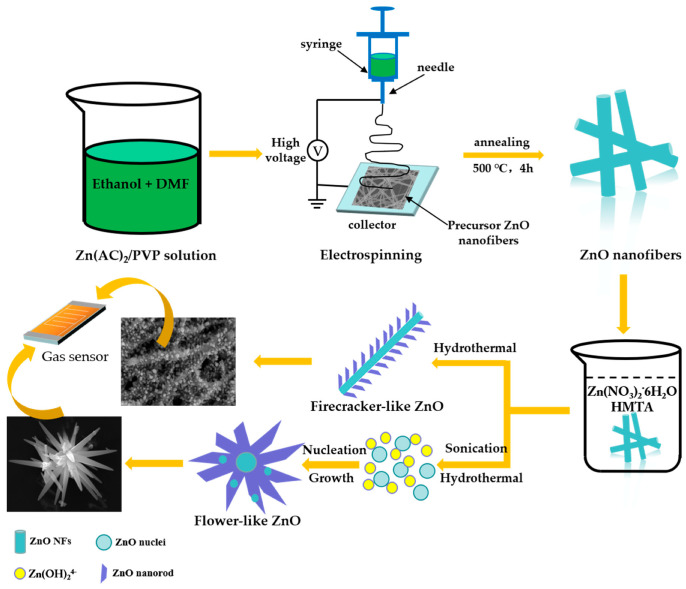
Schematic diagram of the formation of as-prepared firecracker-like ZnO (ZnO HS1) and flower-like ZnO (ZnO HS2) materials.

On the basis of the experiment results, a possible growth mechanism of the ZnO HS1 and ZnO HS2 is illustrated in Figure 6. The specific chemical reactions are as follows [42]:

(CH_2_)_6_N_4_ + 6H_2_O → 6HCHO + 4NH_3_ (1)

NH_3_ + H_2_O ↔ NH_4_^+^ + OH^−^ (2)

Zn^2+^ + 2OH^−^↔ Zn (OH)_2_ (3)

Zn (OH)_2_ + 2OH^−^ ↔ Zn (OH)_4_^2−^ (4)

Zn (OH)_4_^2−^ → ZnO + H_2_O + 2OH^−^ (5)

Generally, ZnO nanorods tended to achieve the crystal growth on the seed layer and existing nucleus [43]. Meanwhile, the ZnO nanoparticles of ZnO NFs could be the seed layer and template to grow ZnO nanorods [39,40]. In the hydrothermal process, with the temperature of the hydrothermal solution increasing, HMTA hydrolyzed to HCHO and NH_3_. Then, NH_3_ reacted with H_2_O to form NH_4_^+^ and OH^−^. Meanwhile, the hydrolyzed Zn^2+^ from Zn (NO_3_)_2_·6H_2_O reacts with OH^−^ in the solution to produce ZnO nanorods under the hydrothermal condition of 80 °C for 12 h. It has been well documented that the growth of ZnO via hydrothermal method consists of a crystal nucleation and a crystal growth stage [37,38,44]. Here ZnO NFs played a seed layer role in the formation of ZnO HS1. A large number of ZnO nanoparticles were present on the surface of ZnO NFs and all these nanoparticles could be the nucleus for the growth of ZnO. Finally, the ZnO HS1s were obtained from the hydrothermal reactions. Besides, the ZnO nanoparticles (Figure S1) generated from the ultra-sounded ZnO nanofibers would become the nucleus for the formation of ZnO HS2. Ultimately, ZnO nanorods would grow around the hybrid nanoparticles, which could lead to the formation of nanoflowers. Next, ZnO HS1 and ZnO HS2 materials were used as the sensing materials of the gas sensors for further gas sensing tests.

### 3.3. Gas Sensing Tests

**Figure 7:**
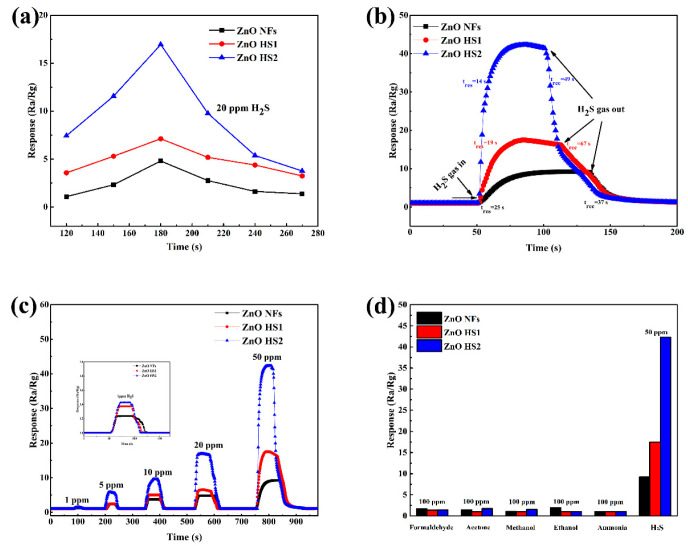
(**a**) Responses of heat-treated ZnO NFs, ZnO HS1 and ZnO HS2 gas sensors toward 20 ppm H_2_S gas at different operating temperatures, ranging from 120 °C to 270 °C. (**b**) Dynamic transient sensing of heat-treated ZnO NFs, ZnO HS1 and ZnO HS2 gas sensors toward 50 ppm H_2_S gas at 180 °C. (**c**) Response-recovery curves of the ZnO NFs, ZnO HS1 and ZnO HS2 gas sensors under different concentrations of H_2_S gas, ranging from 1 to 100 ppm at 180 °C. (**d**) The responses of heat-treated gas sensors based on ZnO NFs, ZnO HS1 and ZnO HS2 toward 50 ppm H_2_S gas and different 100 ppm gases at 180 °C.

The gas sensing performances of ZnO NFs, ZnO HS1 and ZnO HS2 sensors were investigated and the results are displayed in Figure 7. Figure 7a shows the responses of gas sensors toward 20 ppm H_2_S ranging from 120 °C to 270 °C. As the working temperature increased, the responses of gas sensors toward H_2_S gradually improved. At a 180 °C working temperature, all gas sensors obtained the highest response value, but the gas sensors’ responses toward H_2_S decreased above the 180 °C working temperature. Based on these results, further gas sensing tests were carried out at 180 °C. Figure 7b displays the dynamical response transient curves of ZnO NFs, ZnO HS1 and ZnO HS2 sensors upon exposure to 50 ppm H_2_S at 180 °C, respectively. Additionally, the response and recovery time of the ZnO HS2 sensor were 14 s and 49 s, whereas the ZnO NFs and ZnO HS1 sensors exhibited longer response and recovery times of 25 and 37 s, and 19 and 67 s, respectively. More importantly, the ZnO HS2 sensor showed a shorter reply time (14 s) and bigger response (42.298) compared to the other sensors (Figure 7b). Figure 7c presents the dynamic response and recovery curves of gas sensors toward different concentrations of H_2_S. The ZnO HS2 sensor exhibited excellent reproducibility under various concentrations of H_2_S and presented a larger change of response compared with ZnO NFs and ZnO HS1 sensors. Furthermore, the selectivity of gas sensors was also measured. The sensing performances of sensors to different target gases, including 100 ppm ethanol, acetone, methanol, formaldehyde and ammonia, and 50 ppm H_2_S, were investigated. Although the results in Figure 7d reveal that all the gas sensors based on ZnO NFs, ZnO HS1 and ZnO HS2 exhibited good selectivity toward H_2_S gas at 180 °C, the ZnO HS2 sensor showed stronger selectivity toward H_2_S. Overall, the ZnO HS2 sensor exhibited a better selectivity and response performance toward H_2_S. Therefore, it has more potential to be applied in the detection of H_2_S gas.

**Figure 8:**
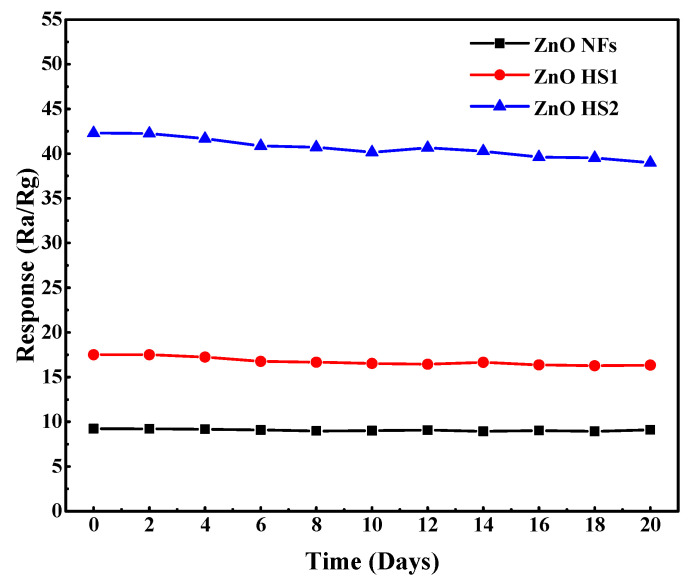
The long-term stability of ZnO NFs, ZnO HS1 and ZnO HS2 gas sensors toward 50 ppm H_2_S at 180 °C.

The long-term stability is also a vital parameter of gas sensors. Figure 8 shows the stability results of three gas sensors toward 50 ppm H_2_S over a term of 20 days. The sensing performance of gas sensors was carried out every two days for 20 days. During 20-days stability tests, the ZnO NFs gas sensors exhibited the best stability, with only a 3% drop in response. However, both ZnO HS1 and HS2 sensors still exhibited a higher response than the ZnO NFs sensor, with 7% decreases in response. Therefore, the ZnO NFs sensor is better in terms of stability, and ZnO HS1 and HS2 sensors are better in terms of response performance.

The comparison results of the sensing characteristics of ZnO gas sensors in the present work with other previous ZnO nanostructures gas sensors are shown in Table 1. The ZnO gas sensors in this work displayed a shorter recovery time than those other reported ZnO gas sensors. ZnO HS2 sensor exhibited shorter recovery time and higher response value compared with other ZnO gas sensors. Therefore, ZnO HS2 material exhibited better H_2_S sensing properties, which has the potential to be applied in the detection of H_2_S gas.

### 3.4. Gas Sensing Mechanism

The change of resistance because of the charge transfers between detected gas and the surface of ZnO samples is generally the sensing mechanism of ZnO based gas sensors [48]. When ZnO sensors were exposed to air at 180 °C, oxygen molecules were absorbed on the surface of ZnO [49]. Meanwhile, the electrons were extracted from the conduction band of ZnO, resulting in the formation of chemisorbed oxygen species O^−^ (100–300 °C) [50,51]. The depletion layer and potential barrier would have formed during the above process, which increased the resistance of sensors. That mechanism could be expressed by the equations [35]:

O_2_ (g) → O_2_(ads) (6)

O_2_ (ads) + e^−^ → O_2_^−^ (ads) (7)

O_2_^−^ (ads) + e^−^ → 2O^−^ (ads) (8)

When the ZnO sensors are exposed to H_2_S, the electrons are returned to the conduction band after the O^−^ reacts with H_2_S. At the same time, the H_2_S molecules react with ZnO and forms zinc sulfide (ZnS). The resistance of ZnO sensors would have an obvious and rapid decrease after the introduction of H_2_S. Owing to the bond energy of H–SH in H_2_S being 381 kJ/mol, lower than ethanol (436 kJ/mol), methanol (436.8 kJ/mol), ammonia (391 kJ/mol) and acetone (393 kJ/mol), breaking the bond H–SH at 180 °C was easier than those other gases. Although formaldehyde (364 kJ/mol) and acetone (393 kJ/mol) and ammonia (391 kJ/mol) also have small bond energies, the reactions between the sensor and target gases were also important. The reaction between ZnO and H_2_S is a spontaneous reaction, as shown in Equation (10), which can increase the sensitivity of ZnO to H_2_S. However, formaldehyde, methanol and ethanol, etc., more easily react with active oxygen species (O^−^), as shown in Equation (11), where the R refers to these gases. The related reactions are as described [20,36,46,52]:

H_2_S(g) + 2 O^−^ (ads) → H_2_(g) + SO_2_(g) + 2e^−^ (9)

ZnO(s) + H_2_S(ads) → ZnS(s) + H_2_O(g) (10)

R(ads)+ O^−^ (ads) → RO + e^−^ (11)

**Figure 9:**
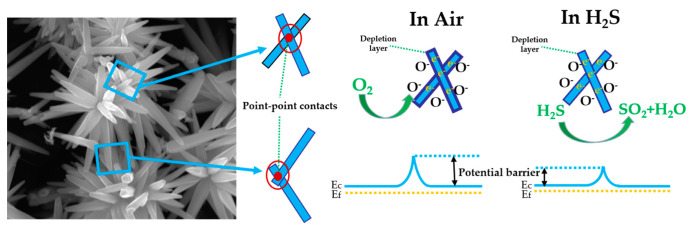
Schematic diagram of ZnO HS2 gas sensitivity mechanism.

Finally, the above process would result in the resistance of ZnO sensors and a decrease in the potential barrier as shown in Figure 9. At the desorption process, the ZnS is converted to ZnO by reacting with O_2_; the reaction is shown as follows [53,54]:

2ZnS(s) + 3O_2_(g) → 2ZnO(s) + 2SO_2_(g) (12)

The reasons for the better gas sensing performance of the ZnO HS2 sensor are described as follows. It can be observed from the Figure 4e,f (SEM) and Figure 5d (TEM) that the ZnO nanoparticles without growing into ZnO nanoflowers were distributed on the surface of the ZnO HS2 and the diameters of nanoparticles were measured to range from about 50 to 150 nm. In previous literature [55], the synergistic effect has been used to improve the sensing response, because more active sites could be generated. Therefore, the ZnO HS2 structures were composed of both nanoparticles and nanoflowers, which could synergistically improve gas sensing properties [56,57,58]. Moreover, owing to the large size of nanorods constituting the ZnO HS2, more point–point contacts existed in the ZnO HS2 sensor. It can be obviously seen from Figure 9 that many point–point contacts were in the ZnO HS2, which could increase the number of potential barriers. To the best of our knowledge, electrons are required to overcome the potential barriers which could result in the higher resistance of the sensor [59,60]. In contrast, the nanorods grown on the surface of ZnO HS1 were smaller in length as shown in Figure 4a–c. The number of point–point contacts was smaller than that of ZnO HS2. The high selectivity of ZnO HS2 may be attributed to the morphology of nanorod bundles of ZnO HS2 and lower H–SH bond energy [61,62].

## 4. Conclusions

In summary, both firecracker-like and flower-like ZnO hierarchical structures have been successfully fabricated without using surfactants. ZnO HS2 displayed the morphology of nanoflowers modified by nanoparticles. The growth process diagram of firecracker-like and flower-like ZnO hierarchical structures was described. The gas sensing results revealed that gas sensors exhibited high selectively and good long-term stability toward H_2_S. The gas sensing studies demonstrated that the ZnO HS2 gas sensor exhibited the highest sensing properties toward H_2_S. The ZnO HS2 gas sensor has a much higher response toward H_2_S gas (50 ppm H_2_S, 42.298) than the ZnO NFs gas sensor (50 ppm H_2_S, 9.223) and ZnO HS1 gas sensor (50 ppm H_2_S, 17.506). Systematic sensing tests of gas sensors toward H_2_S, including different temperatures and different concentrations were carried out, and the gas sensing mechanism of the sensors was also proposed. The synergistic effect of nanoparticles and nanoflowers, and more point–point contacts between nanorods of ZnO HS2 are advantageous for the high H_2_S sensing properties of flower-like ZnO hierarchically structured material. Our study indicated that the morphology of ZnO materials affect the gas sensing properties of materials, and that hierarchically structured ZnO materials could be developed as inexpensive and excellent H_2_S gas sensors.

## Figures and Tables

**Table 1:** Comparison of sensing characteristics of different ZnO nanostructures gas sensors.

Materials	H_2_S (ppm)	Response	Response Time	Recovery Time
ZnO NFs *	50	9.223	25 s	37 s
ZnO HS1 *	50	17.506	19 s	67 s
ZnO HS2 *	50	42.298	14 s	49 s
ZnO nanosheet [5]	100	23	252 s	3697 s
ZnO thin film [45]	100	3.2	10 s	198 s
ZnO nanorods [46]	100	63	4 s	60 s
ZnO nanostructures [47]	20	80	35 s	390 s

* Refers to ZnO gas sensors in this work.

## Supplementary Materials

The following are available online at https://www.mdpi.com/2079-4991/9/9/1277/s1, Figure S1: SEM image of the ultra-sounded ZnO NFs; Figure S2: SEM images of as-prepared (**a**) ZnO HS1 sample and (**b**) ZnO HS2 samples after application in the gas sensing.
